# Sexual violence perpetrated by health professionals

**DOI:** 10.3389/fpsyg.2022.1005696

**Published:** 2023-04-03

**Authors:** Marisalva Fávero, Vanessa Gomes, Amaia Del Campo, Diana Moreira, Valéria Sousa-Gomes

**Affiliations:** ^1^Social and Behavioral Sciences Department, University of Maia, Maia, Portugal; ^2^Justice and Governance Research Center of the Law School, University of Minho (JusGov/UM), Braga, Portugal; ^3^Department of Evolutionary and Educational Psychology, University of Salamanca, Salamanca, Spain; ^4^Institute of Psychology and Neuropsychology of Porto – IPNP Health, Porto, Portugal; ^5^Laboratory of Neuropsychophysiology, Faculty of Psychology and Educational Sciences, University of Porto, Porto, Portugal; ^6^Centro de Solidariedade de Braga/Projecto Homem, Braga, Portugal

**Keywords:** sexual violence, health professionals, direct victims, indirect victims, rape

## Abstract

Sexual violence (SV) perpetrated by health professionals concerns any sexual conduct, whether physical or verbal (with or without contact), toward a patient. There has been little scientific study and some disagreements have emerged regarding its definition, which has even been confused with violation of professional boundaries. In this descriptive-exploratory study, we aimed to characterize this phenomenon in the Portuguese context, using a sample of 491 participants who completed an online questionnaire adapted for this study. The results showed that 8.96% of the participants (5.5% indirect victims) suffered SV by a health professional, and the sociodemographic characteristics are very similar to those of SV in other contexts. Thus, after confirming this is not a problem alien to the Portuguese reality, we discuss the practical implications for prevention and intervention with victims.

Sexual violence (SV) is a social and public health problem (De Freitas and Farinelli, 2016), since it goes beyond the private domain and leads to consequences in several areas of the victim’s life (Fávero et al., 2021). According to the World Health Organization [WHO] (2002), SV is “any act that results in, or is likely to result in, physical, sexual, or psychological harm or suffering, including threats of such acts, coercion or arbitrary deprivation of liberty, whether occurring in public or in private life” (p. 149). It also pertains to “(…) any sexual act, attempt to obtain a sexual act, or other act directed against a person’s sexuality using coercion, by any person regardless of their relationship to the victim, in any setting” (Costa de Souza et al., 2013, p. 99). SV is a very current issue and has been a subject of interest for several researchers (e.g., Blechner, 2014; Alpert and Steinberg, 2017; Fávero et al., 2021).

However, few studies focus on SV perpetrated by health professionals. Thus, there is a need for studies on SV experienced in settings where the victim seeks professional help, whether in terms of physical or mental health. Most studies on this issue have been conducted in the USA (Melville-Wiseman, 2012). Therefore, the present research aims to fill this gap by providing a detailed characterization of this phenomenon in the Portuguese context.

Before moving on to the reflection about SV in the health context, it is important to mention that the issue of sexual interactions between professionals and clients has been approached in two ways. Some studies analyze these sexual interactions as a violation of the boundaries of professional relationships (Gutheil and Gabbard, 1993; Plaut, 1997; Blechner, 2014). Other studies analyze the phenomenon as SV, which is the approach used in the present study. As such, we will initially clarify these two concepts by making a brief distinction between violating professional boundaries and SV, since there is some confusion between the two.

Studies that approach SV by professionals as if it were about violating the boundaries, on one hand, focus on studying boundaries, rather than framing the data as SV (Gutheil and Gabbard, 1993; Plaut, 1997; Blechner, 2014). However, even if they are consensual, sexual interactions in the context of professional relationships, between a professional who provides a service and a client, violate ethical and deontological principles (Blechner, 2014; APA, 2018). On the other hand, SV refers to any sexual conduct, whether verbal or physical, toward the patient, or something that can be interpreted as such, which is non-consensual or obtained under coercion (DuBois et al., 2019).

Therefore, there is an inconsistency in the studies that frame these acts as violations of professional boundaries and not as SV (Gutheil and Gabbard, 1993; Plaut, 1997; Blechner, 2014). According to Plaut (2008), violations of professional boundaries can be distinguished in three ways: sexual harassment (e.g., sexual allusion), misconduct (e.g., therapist-patient intimacy), and non-sexual dual relationships (e.g., exchanging gifts). The author also emphasizes that these situations can start at an early age, between teacher and student, leading to a bad example and poor practices in the future as a professional. Thus, according to the author, harassment is seen as a breach of boundaries and not as SV, which contradicts the very definition of SV. Therefore, some researchers argue there are boundary violations that are not of a sexual nature, which are the most common, such as the exchange of gifts or invitations to dinner. However, studies on SV have concluded that these are often the precursors to rape (Alpert and Steinberg, 2017).

In general, the perpetrators of SV are mostly men, and women are victims. This is also observed in health contexts, where the perpetrators are mostly men in 80% of the cases (Schoener et al., 1989) and the victims are mostly women (Pope, 1994; Garrett, 2002). Only exceptionally do women commit violations of this nature, and when they do, it is with people of the same sex (Garrett, 2002).

Studies conducted in the United States of America and Canada in the 1990s, on SV in the health field, found that about 10% of psychotherapists got involved with a client throughout their professional career. Regarding doctors from other fields (unrelated to mental health), the reality is similar, with approximately 9% of them having sexual contact with at least one patient (Gartrell et al., 1992).

Recently, one of the few prevalence studies, conducted using reports from professionals, found that about 16% of male professionals and 3% of female professionals confessed to became sexually involved with their clients (Pope et al., 1979; Borys and Pope, 1989). Another study, with a sample of 1,000 clinical psychologists, revealed that 3.6% of them had already had sexual contact with a patient. In addition, 22.7% of the participants stated they had treated patients who were victims of SV by a mental health professional. Moreover, 38% of participants revealed knowing a psychologist who sexually abused a patient (Melville-Wiseman, 2012).

Data collected from 44 mental health institutions revealed 300 cases of SV during a period of 3 years (between 2003 and 2006) (Melville-Wiseman, 2012). Of these, about one third were perpetrated by employees. It is important to mention this data was made available by the managers of the institutions, and may not represent the full extent of the phenomenon. Overall, it was possible to perceive a feeling of shame and powerlessness in the face of abusive sexual behavior perpetrated by some of their colleagues and many do not report it for fear of suffering retaliation (Melville-Wiseman, 2012).

In fact, the issue of sexual assault by professionals has been the subject of scientific research and professional concerns in the fields of medicine, psychiatry, psychology, among others, for decades, as evidenced by some of the aforementioned studies. Some authors believe attention should be paid to the boundaries established in the different contexts where SV occurs, especially in the fields of mental health, since these contexts, when compared to some medical ones, are more prone to SV, which is mainly due to the mental vulnerability of the person seeking help. However, there are also medical contexts that should have better defined ethical and deontological boundaries than other contexts, for example, when we compare a gynecologist with a radiologist. The gynecologist maintains a more intimate contact with the patient, making sexual assault easier to commit and harder to prove (Gutheil and Gabbard, 1993; Smith and Fitzpatrick, 1995).

According to research conducted by Sansone and Sansone (2009), most SV in medical contexts occurs in family planning, psychiatry, obstetrics, and gynecology consultations. The high prevalence in these areas is justified by the greater proximity/contact between the patient and the professional in these specific environments. The authors also found that most perpetrators are male (85%) and that only about 1.6% of perpetrators were punished for the crime they committed, although approximately 7% reported sexual relations with patients. According to the researchers, many of these sexual crimes are not punished, because they are omitted by the context in which they are perpetrated (Reling et al., 2018).

Studies show that the setting of the psychotherapeutic relationship is an environment conducive to SV by professionals and that the silence of the victim is mainly due to the transference process (patient-therapist) that occurs due to the existence of unresolved (external) problems; thus, the patient becomes more receptive to sexual behavior and experiences a weaker capacity to report it (Alpert and Steinberg, 2017).

Furthermore, within the areas of mental health, namely in the area of psychology, there are theoretical models that allow for greater proximity with the patient. For example, a psychoanalyst may consider an out-of-office consultation inappropriate, whereas a behaviorist may request the context/environment as a therapeutic setting (Gutheil and Gabbard, 1993; Smith and Fitzpatrick, 1995), and the psycho dramatists use physical contact in specific situations in the dramatization stage (Hudgins, 2022).

Compared to therapist-patient relationships whose boundaries are better defined, nurse-patient relationships are marked by more proximity and physical contact; thus, there is more risk of the professional abusing the patient (Bachmann et al., 2000). However, the literature on SV between nurse-patient is almost non-existent and, therefore, a field of investigation to be considered by the scientific community in future studies.

Regarding the doctor-patient relationship, some studies argue this is a profession that requires a lot of proximity, which sometimes makes it difficult to differentiate the roles of each of those involved, with a comparison even being made between this relationship and intrafamily relationships. Therefore, the existence of similarities between the doctor-patient relationship and intrafamily relationships suggests a greater propensity for sexual relationships (Bachmann et al., 2000). According to Bachmann et al. (2000), these sexual relationships can serve as a model for intrafamily sexual relationships, which can lead the patient to legitimize the SV. In this context, SV is more often perpetrated in inpatient settings (hospitalization) than in outpatient settings.

Social work is among the professions in which SV is more likely to take place, since conflicts of interest are very common, due to involvement with clients in different areas of life, namely social, work, among others (Reamer, 2003). According to different studies, a significant part of relationships with social workers involves sexual relationships (Reamer, 2003).

SV is a strong constraint on physical and mental wellbeing and health, which is why it is a subject of great importance for research (Krahé, 2016). In addition, victims of SV may have sexually transmitted diseases, for example Hepatitis B or HIV, and, in more severe cases, unwanted pregnancy and Post-Traumatic Stress Disorder (Drezett, 2014). Post-Traumatic Stress Disorder results from exposure to traumatic experiences, in this case SV, resulting in feelings of anxiety, excessive irritability or apathy, difficulty concentrating and sleeping, and avoiding people or places that reactivate traumatic episodes (Wies and Coy, 2013).

In a study by Eichenberg et al. (2010), patients claimed it was the therapists who initiated sexual contact. In general, 86.5% of the sample under study exhibited negative results, with therapists causing serious damage in the lives of their patients. The findings emphasize different feelings and symptoms precipitated by the SV, including “(…) mistrust, isolation, feelings of shame and guilt, fear, depression and suicidal tendencies, anger and symptoms of post-traumatic stress disorder” (Eichenberg et al., 2010, p. 1,019).

The harm caused by sexual assault is not limited to the aforementioned effects. It also includes anxiety, addiction, regression, and depersonalization. In addition, uncontrolled spending, poor time management, as well as recollection of some previously repressed memories/feelings are often present (Hook and Devereux, 2018).

Thus, regardless of the strategy and/or behavior used by the perpetrator during the SV, it causes significant harm to both the patient and the therapeutic relationship (Alpert and Steinberg, 2017).

Pioneer studies from the 1990s (Plaut, 1997) have already corroborated the current idea that most cases of SV are not disclosed by clients/patients (Krahé, 2016). The justification for this fact is the possible discrediting of the complaint by the person who receives it. In addition, Krahé (2016) points out the almost non-existence of civil liability insurance covering sexual exploitation. Moreover, the committees responsible these types of situations do not have a good repertoire regarding the execution of justice in these cases (Ventura, 2018).

The fact that SV does not always start with more obvious sexual behavior, such as sexual intercourse with vaginal or anal penetration, also makes it difficult for the victim to report it (Fávero et al., 2022). Sometimes, SV occurs in the form of masturbation in front of patients or through contact with the genitals, among others. In other words, it starts in a more subtle way and only when it becomes more evident does the victim realize it and, then, feels simultaneously ashamed and afraid that people will question them as to why they did not notice the situation before and report it (DuBois et al., 2019). This is a reality most likely hidden by the judgment and stereotypes surrounding victims, not only by common sense, but also by the justice system (Krahé, 2016). The fact that victims of SV are confronted daily with negative social attitudes makes them feel more reluctant to disclose the event. When they feel able to do so, they experience a new process of victimization potentiated by third parties—secondary victimization (Krahé, 2016), which can lead to a decrease in self-esteem and emotional commitment (Costa de Souza et al., 2013). As in other contexts, most cases of SV remain hidden, thus there is no way to punish the perpetrator (Krahé, 2016).

Therefore, the question arises: how many cases are yet to be discovered? There are people who simply think sexual intercourse brings therapeutic benefits, and others who do not even perceive the situation as SV (Dahlberg, 2014). Thus, in order to avoid SV, it is important to be aware of the possibility of transference and countertransference, as well as have tools to deal with them. Above all, there must be good supervision and a needs assessment of the professionals applying for the job (Faulkner and Regehr, 2011). According to Lazarus (1994) and Galletly (2004), an inadequate management of these personal dilemmas can compromise the relationship between the therapist and the patient, as well as the results of the therapy. Finally, our analysis of the existing literature allows us to conclude that researchers had little interest in the characterization of this phenomenon. As stated by Dahlberg (2014), this is a known topic among other researchers, however, it has been left out of the literature for a long time.

Aware of this reality, professionals of the field have proposed programs aimed at preventing SV by professionals, including the “Professional Boundaries for Health Professionals (PBHP)” program, which addresses real ethical dilemmas faced by health professionals. They must decide the best course of action for dilemmas that might be experienced in different work contexts. The codes of ethics of different professional fields are also discussed to raise awareness among health professionals (Fronek et al., 2009).

However, throughout this review, it was possible to verify that SV occurs in professional service provision in the field of health, in which the perpetrator is the provider of this service (Abrahams et al., 2014). However, there are almost no studies with Portuguese samples.

These studies cited in the review were identified through search on EBSCO, PubMed, and Web of Science. The reference lists of the selected studies were also reviewed to identify other relevant studies. The studies were considered for analysis if they met the following inclusion criteria: (a) studies that have a SV/harassment (b) committed by health professionals. As exclusion criteria, three categories were used: (a) wrong theme (articles that do not include SV committed by health professionals); (b) wrong population (sample without health professionals); (c) wrong outcome (articles that do not include measures of SV). The search was not limited by any geographical, temporal, or age factors. Duplicate articles were eliminated. The studies were selected by two independent reviewers, based on their titles and abstracts, according to recommendations of PRISMA guidelines (Moher et al., 2010).

Therefore, the present retrospective study, of a descriptive-exploratory nature and with impact assessment, aims to contribute to the understanding and characterization of the phenomenon of SV perpetrated by health professionals in the Portuguese context. Specifically, we intend to (1) analyze the prevalence, frequency, and duration of SV; (2) characterize the perpetrators and victims, namely, age and strategies/behaviors adopted in the case of SV; (3) explore the health contexts most conducive to this violence; and, finally, (4) examine the consequences of this phenomenon on victims, both in terms of symptoms and reporting/disclosure.

## Materials and methods

### Participants

Participants in the present study were randomly selected by completing an online questionnaire. The inclusion criteria were to be at least 18 years of age and having resided in Portugal or the sexual assault having occurred in Portugal. The sample selection method used was convenience sampling. The study was mainly disseminated on the research team’s online social networks, as well as in their social circles (e.g., acquaintances, friends, family). The only inclusion criteria were the minimum age of 18 years and residing in Portugal. The sample consists of 491 individuals, with an average age of 26.47 years and 85.1% of which were female (*n* = 418). Most of the participants lived in the North of Portugal 44.6% (*n* = 219), followed by the South 29.7% (*n* = 146), Lisbon Metropolitan Area 15.7% (*n* = 77), the Center 6.5% (*n* = 32), Autonomous Region of Madeira 2.4% (*n* = 12), Autonomous Region of Azores 0.8% (*n* = 4) and, finally, Alentejo with 0.2% (*n* = 1). Regarding marital status, most participants were single 42.4% (*n* = 208), 27.7% (*n* = 136) reported being in a committed relationship but without living together, 12.4% (*n* = 61) were in a *de facto* union, 11% (*n* = 54) were married, 3.3% (*n* = 16) mentioned being in a non-committed/occasional relationship, 2.6% (*n* = 13) were divorced and, finally, 0.6% (*n* = 3) were separated. As for nationality, most participants, 97.5% (*n* = 476), were Portuguese, 0.8% (*n* = 4) were Brazilian, 0.4% (*n* = 2) were German and, lastly, 0.2% belong to each of the following nationalities: Angolan, Guinean, Russian, Irish, Swiss, and Romanian, corresponding to one person of each nationality.

Regarding the level of education, 42% have secondary education (grades 10–12) (*n* = 206), followed by 34.6% with a post-secondary degree *in* = 170, 7.7% with a master’s degree (*n* = 38), 5.9% have a technological specialization course (*n* = 29), 5.7% have the 3rd cycle of basic education (grades 7–9) (*n* = 28), 2.6% with a bachelor’s degree (*n* = 13), 1% with a doctoral degree (*n* = 5) and, finally, 0.4% have the 2nd cycle of basic education (grades 5 and 6) (*n* = 2).

Finally, considering the main activity of the participants, 48.6% are students (*n* = 238), 45.5% are workers (*n* = 223), 5.5% are unemployed (*n* = 27), 2% mention not having a profession (*n* = 1), and 2% are workers on sick leave (*n* = 1).

### Instruments

For this study, we used a questionnaire consisting of three instruments, which was made available online through the Google Forms Platform.

1. Questionnaire for the Assessment of Sexual Assault in Professional Relationships (Fávero et al., 2010), composed of four parts: (1) Pertains to the sociodemographic data of the participants; (2) Refers to the legitimation of sexual assault in professional relationships and the perception that participants have of professional boundary violations; (3) Collects data on direct experiences of victimization of SV perpetrated by professionals and (4) Collects information related to the witness, that is, about having indirectly witnessed/experienced a situation of SV by health professionals.

The questionnaire was developed by elements of the research team, and it is a comprehensive questionnaire with a sociodemographic characterization of the sample. Complete open-ended questions (e.g., “In which city did the sexual assault take place?”), closed-ended questions [e.g., “Throughout your life, have you ever been a victim of SV by a health professional (doctor, psychologist, physical therapist, physiatrist, etc.)? (SV is understood as any contact or non-contact behavior) * Yes/No], and multiple-choice questions (e.g., “What is the duration of sexual assaults?” * (tick only the most frequent: (a) Never happened again; (b) On the same day; (c) In the same week; (d) During a month; (e) Between 2 and 6 months; (f) Between 6 months to a year; (g) 1 or 2 years; (h) More Than 2 years).

2. SRQ-20—Self Reporting Questionnaire (OMS), which aims to screen for the diagnosis of mental disorders, through 20 items based on the criteria of the DSM—Diagnostic and Statistical Manual of Mental Disorders. The responses are yes/no and each affirmative answer has a score of 1. The final classification is produced by summing up the total scores obtained, with zero representing no probability for mental disorder and 20 representing a high probability for mental disorder (Gonçalves et al., 2008). Internal consistency was examined using Cronbach’s Alpha. The original internal consistency is 0.86. The value obtained in the present study is 0.839, which indicates good internal consistency.

3. Depression Anxiety and Stress Scale (DASS-21, Lovibond and Lovibond, 1995, adapted to the Portuguese population by Pais-Ribeiro et al., 2004), comprises three subscales about anxiety, depression and stress, each with seven items. It has 21 items with four response options, on a Likert-type scale, where individuals evaluate the extent to which they have experienced each symptom over the past week. The original internal consistency, examined using Cronbach’s Alpha, was 0.85 for the depression scale, 0.74 for the anxiety scale and 0.81 for the stress scale. The results of the present study were 0.859 for the depression scale, 0.867 for the anxiety scale, and 0.898 for the stress scale. Total Cronbach’s Alpha was 0.949. Thus, the instrument used reveals good internal consistency, both globally and for each of its factors.

### Procedures

The questionnaire was made available online on the Google Forms Platform. We used convenience and geometric propagation sampling (snowball) to supplement relevant data for the study. We obtained a total of 509 participants, 18 of which were eliminated, because they did not meet the inclusion criteria.

All confidentiality procedures and ethical and legal recommendations were guaranteed, and informed consent was requested for the voluntary collaboration of the participants. In addition, the anonymity and confidentiality of the data were guaranteed. This study was approval by the Ethics Committee of the University of Maia (CED 101/2022). The data was imported into a statistical analysis program—IBM Statistical Package for the Social Sciences (SPSS), version 24.0, and then analyzed. The characterization and description of the study sample, and respective variables, was performed using frequency and descriptive statistics.

## Results

The present study aimed to understand and characterize SV perpetrated by health professionals, in the Portuguese context. The results will be detailed for each specific objective separately.

Considering that the main objective is to analyze the prevalence of sexual assault in professional health contexts in Portugal, through descriptive statistics, it was possible to confirm that, of the 491 participants, 44 claimed to have suffered SV by a health professional (8.96%). Of these, 3.5% (*n* = 17) are direct victims, that is, participants who suffered SV, and 5.5% (*n* = 27) reported knowing a victim in this context (whom we will refer to as witnesses).

Regarding witnesses, it is also possible to detail the victims to whom these participants refer. Specifically, 85.2% to a friend (*n* = 23), 7.4% refer to their mothers (*n* = 2), 3.7% to their aunts (*n* = 1), and 3.7% to a colleague (*n* = 1). As for the frequency of the sexually violent behaviors, 87.5% (*n* = 14) of the victims went through this situation once and 12.5% (*n* = 2) up to three times. Regarding the duration, 88.2% (*n* = 15) said it never occurred again, 5.9% (*n* = 1) said it occurred during 1 month and 5.9% (*n* = 1) said it happened between one and 2 years. It should be noted that, since victims considered the problem to be common, they suggested some recommendations to help prevent and/or end it, as well as provided information for a better understanding of the cases. In particular, they suggested there should be better information on the technical procedures used by health professionals (37.5%, *n* = 3), more investigation should be conducted on hospitals (12.5%, *n* = 1), cameras or recorders should be installed in the offices (12.5%, *n* = 1), society’s ideas on the subject should change (12.5%, *n* = 1), never be suspicious of the victim (12.5%, *n* = 1) and difficulty in reporting may be related to the perpetrator’s social status (12.5%, *n* = 1).

The second objective was to understand which sex was predominant among perpetrators. The results showed that, in 41 cases, the perpetrator was male (93.18%) and, in three cases, the perpetrator was female (6.82%). Regarding the perpetrators’ characteristics, 45.45% were between 31 and 50 years of age (*n* = 20), 38.63% were over 50 years old (*n* = 17), 11.36% (*n* = 5) were between 21 and 30 years of age, and 2.28% (*n* = 1) were between 18 and 20 years of age. The remaining 2.28% (*n* = 1) refer to an omitted case, in which the witness did not have the information.

Regarding the abusive behavior, the direct victims reported caresses and/or kisses to their breasts (36.4%, *n* = 4), caresses and/or kisses to other parts of the body (27.3%, *n* = 3), body contact with hugs and prolonged kisses (lips and tongue) (18.2%, *n* = 2), caresses and/or kisses on their genitals (18.2%, *n* = 2), invitation to some type of sexual activity (9.1%, *n* = 1), exhibitionism (9.1%, *n* = 1) and oral sex (9.1%, *n* = 1). Some victims added other behaviors by the perpetrators, namely nudity (50%, *n* = 3), uncomfortable positions (33.3%, *n* = 2) and inappropriate touching (16.7%, *n* = 1).

As for the witnesses, they mentioned caresses and/or kisses on the breasts (30.8%, *n* = 4), caresses and/or kisses on other parts of the body (23.1%, *n* = 3), body contact with hugs and prolonged kisses (lips and tongue) (15.4%, *n* = 2), caresses and/or kisses on genitals (15.4%, *n* = 2), exhibitionism (7.7%, *n* = 1) and oral sex (7.7%, *n* = 1). Witnesses also mentioned other behaviors, namely nudity (37.5%, *n* = 3), excessive force used in childbirth (12.5%, *n* = 1), sexual conversations *via* telephone (12.5%, *n* = 1) and verbal innuendo (12.5%, *n* = 1).

Victims indicate that the main strategies used by perpetrators were approach/surprise (31%, *n* = 9), taking advantage of trust or familiarity (24.1%, *n* = 7) and their own authority (20.7%, *n* = 6), deceit (13.8%, *n* = 4), use of force (3.4%, *n* = 1), use of force with harm (3.4%, *n* = 1), and seduction (3.4%, *n* = 1).

Regarding the sex of the victims, there was a total predominance of females, that is, of the 44 cases revealed, all the victims were female (100%).

As for the age at which the sexual assault took place, the victims were between 18 and 47 years old, with an average age of 26.82 reported by direct victims (mode: 20 years old), and 28.81 reported by the witnesses (mode: 18 years old).

Concerning the behavior adopted by the victim when the SV occurred, 31.3% (*n* = 5) of the victims did not react, 31.3% (*n* = 5) physically resisted the situation the entire time, 25% (*n* = 4) initially did not react, but then resisted and refused, 6.3% (*n* = 1) collaborated from the beginning and 6.3% (*n* = 1) left the location.

As for the field of health in which the perpetrator provided services, there is a predominance of general medicine, with 65.91% (*n* = 29). Other specific areas of medicine were mentioned, such as gynecology (9.09%, *n* = 4), obstetrics (4.55%, *n* = 2) and ophthalmology (2.27%, *n* = 1), as well as other health areas such as physiotherapy (6.82%, *n* = 3), nursing (2.27%, *n* = 1), acupuncture (2.27%, *n* = 1), psychology (2.27%, *n* = 1) and social work (2.27%, *n* = 1). In this percentage, there was also an omitted case, which referred to a witness who was unable to specify the health field in which the SV occurred.

Regarding the feelings of direct victims toward the perpetrator, disgust was the most frequently mentioned (51.7%, *n* = 15), followed by outrage (27.6%, *n* = 8), anger (10.3%, *n* = 3), sadness (3.4%, *n* = 1), fear (3.4%, *n* = 1), and distrust (3.4%, *n* = 1). Witnesses mentioned disgust (31.1%, *n* = 19), outrage (27.9%, *n* = 17), anger (19.7%, *n* = 12), distrust (8.2%, *n* = 5), fear (4.9%, *n* = 3), hurt (3.3%, *n* = 2), betrayal (3.3%, *n* = 2), and sadness (1.6%, *n* = 1).

Regarding the feelings related to the experience of SV, there is a great similarity to those mentioned above, namely disgust (34.3%, *n* = 12), shame (17.1%, *n* = 6), helplessness (14.3%, *n* = 5), distrust (11.4%, *n* = 4), hostility or aggression (11.4%, *n* = 4), anxiety/distress (5.7%, *n* = 2), guilt (2.9%, *n* = 1), and fear (2.9%, *n* = 1).

With respect to the immediate effects of the traumatic event that are still present, and which the victims admit trigger the victimization experience, we found feelings of outrage, anger and disgust (29%, *n* = 9), a perception that one cannot count on the people who should provide care (12.9%, *n* = 4), a loss of confidence in most people (12.9%, *n* = 4), a feeling that there is no point in telling anyone about problems (9.7%, *n* = 3), a perception that the blame for SV falls on the victim (9.7%, *n* = 3), learning that in a situation of SV the victim has no power to fight (6.5%, *n* = 2), a change in the relationship with oneself (6.5%, *n* = 2), hypersensitivity and hyperactivity in situations of violence and injustice (6.5%, *n* = 2), difficulty in establishing intimate relationships with an affective partner (3.2%, *n* = 1), as well as sleep and/or eating disorders (3.2%, *n* = 1).

As long-term consequences, we found that victims had difficulty trusting other people (30%, *n* = 3), fear of getting involved in sexual relationships (10%, *n* = 1), difficulty in establishing intimate relationships with an affective partner (10%, *n* = 1), excessive fears (10%, *n* = 1), very anxious reactions (10%, *n* = 1), feelings of guilt (10%, *n* = 1), low self-esteem (10%, *n* = 1) and depression (10%, *n* = 1).

Regarding the consequences in everyday life, we found relocating to another city (50%, *n* = 3), moving house (16.7%, *n* = 1), as well as moving to another workplace (16.7%, *n* = 1) or university (16.7%, *n* = 1).

According to the victims, 64.7% (*n* = 11) mention that SV had “some importance”, followed by “very important” (17.6%, *n* = 3), “somewhat important” (11.8%, *n* = 2) and “not important” (5.9%, *n* = 1).

Regarding the complaint, it was found that 94.1% (*n* = 16) of direct victims did not file a complaint with the police, 5.9% (*n* = 1) reported the perpetrator and the case went to court, but it was cleared. As for the witnesses, it was found that most (85.1%, *n* = 23) of the victims did not file a complaint with the police, 14.8% (*n* = 4) filed a complaint and, of these, 50% (*n* = 2) revealed that the case went to court and the perpetrator was convicted. Concerning disclosure, we found that most victims (47.1%, *n* = 8) disclosed the violence to a friend, 23.5% (*n* = 4) did not disclose it to anyone, 11.8% (*n* = 2) disclosed it to another family member, 5.9% (*n* = 1) to the father, 5.9% (*n* = 1) to a co-worker and 5.9% (*n* = 1) to an administrative assistant of the location where the sexual assault occurred. As for the time it took for the person to disclose the traumatic situation, most victims (50%, *n* = 8) disclosed it on the same day or the next, 25% (*n* = 4) never disclosed it, 18.8% (*n* = 3) disclosed it more than 1 month later and 6.3% (*n* = 1) after 1 year. Regarding the help they received after disclosure, 52.9% (*n* = 9) of the victims said they did not need help, 29.4% (*n* = 5) of the victims revealed that no one tried to help, 5.9% (*n* = 1) revealed having had support from friends, 5.9% (*n* = 1) were taken to a health professional and 5.9% (*n* = 1) got help to replace the offending professional. Finally, in terms of the effectiveness of the help received, 13.3% (*n* = 2) said it was very effective, 13.3% (*n* = 2) mentioned it was somewhat effective, 26.7% (*n* = 4) stated it was not at all effective, 26.7% (*n* = 4) said they did not need help and 20% (*n* = 3) did not get any help.

## Discussion

The present study aimed to understand and characterize SV perpetrated by health professionals in a sample of Portuguese participants. The data indicates the existence of SV in this context, with almost 10% of the participants being victims. The findings point to international prevalence, similar to what was found in the studies by Alpert and Steinberg (2017) on SV in the health context, as well as in studies other contexts (Barth et al., 2013; Gewirtz-Meydan and Finkelhor, 2019; Fávero et al., 2022; Sousa-Gomes et al., 2022). This is evidence that SV is a problem that affects all societies, all contexts, and is not restricted by geographic or socioeconomic factors (Moreira et al., 2021). There is an increasing number of complaints regarding this type of violence, thus it is important to invest in the education of students in the fields of Health. We found that perpetrators are predominantly male and victims are mostly female, thus once again confirming previous studies (Sansone and Sansone, 2009) and configuring SV indeed as a gender offense.

Although the literature review on SV perpetrated by professionals is scarce, the scientific community has focused mostly on sexual aggression perpetrated by physical and mental health professionals, and this can be explained by the greater proximity and intimacy that professionals in these areas establish with patients (Sansone and Sansone, 2009). The present study showed that, in the professional categories within the health field, there is a predominance of SV in medicine, with emphasis on gynecology and physical therapy. Regarding age, as identified in other studies (Dahlberg, 2014), the perpetrators are approximately 50 years old or older, and the victims are an average of 28 s old.

The typical sexual behaviors of perpetrators range from physical touch, mainly from casual kissing to caressing genitals, as well as verbal behavior (without contact), namely sexual phone calls or invitations to activities of a sexual nature, similar to what is observed in SV for this and other contexts. Such information leads us to the idea that participants consider both physical and verbal (non-contact) sexual behaviors to be inappropriate, in line with the professional and legal concept of SV.

The present study found that SV occurred a maximum of up to three times with the same perpetrator. However, for most victims, the SV was a single episode. Although there is not a high percentage of repeated sexual aggression, this type of violence causes a strong impact on the victim, both in terms of symptoms and consequences (Moreira et al., 2021). Indeed, SV causes significant impact on victims, both in the short and long terms, in their daily lives, their social lives, in the way they view themselves, as well as in their belief in themselves and in others. The victims pointed to several short- and long-term consequences, such as disgust, outrage, anger, shame and powerlessness. These sentiments are in line with what is found in existing literature, namely the studies by Eichenberg et al. (2010) and Hook and Devereux (2018). Consequently, participants also suffered some changes in their daily lives, namely moving to another city, difficulty in trusting other people, fear of having sex and difficulty in establishing intimate relationships with a romantic partner.

The results of the current study suggest this is a reality present in Portugal, with very similar characteristics to those found in international reviews. Thus, there is a need for better preparation of health professionals, as well as of the criminal police forces, in order to prevent and intervene in this type of crime.

Furthermore, we concluded that some victims have a strong knowledge of the concept of SV, which is extremely important in order to recognize the situation of sexual aggression and report it quickly. Nonetheless, some victims in this study do not attach much importance to the experience, which may explain the small number of complaints filed with the police, as well as disclosures.

Despite the short- and long-term consequences of SV, with important repercussions on daily life, such as moving house as a result of the abuse, many victims attributed little importance to them. This may explain the low number of complaints filed. However, overall, literature on SV reveals that the reasons for low reporting and disclosure relate to a number of issues.

The lack of disclosure and complaints filed for SV is a constant in the literature, which shows that most victims do not reveal the experience, and complaints and subsequent trials for crimes of this nature are even less frequent. Related to the low number of complaints and disclosure was the fact that victims had lost trust in other people, as well as the fear and shame of being discredited by the person to whom they disclose the crime. This fact is also pointed out by Plaut (1997) and Ventura (2018), who mention that the ethics committees that assess these situations do not have a good repertoire with regard to the execution of justice in this type of case, which may also influence this attitude by the victims. At the same time, the frequency and duration of SV also influence the reporting/disclosure and symptoms/consequences for the victim.

The present study allowed us to deepen the knowledge about SV and alerted to the serious gaps that exist regarding this subject, which were explained throughout the literature review. In particular, we mentioned the lack of research on SV perpetrated by health professionals and the consequent lack of information for better prevention and action with regard to this problem. In addition, not only is the literature scarce, but the existing studies are also old, which hinders the possibility of having a current view of the phenomenon. Even so, these studies made it possible to draw some conclusions about the transversal characteristics among cases of SV perpetrated by health professionals, namely with regard to perpetrators and victims, as well as the impact caused in various areas of the victims’ lives. Overall, it was found that all victims had/have some type of impact, to varying degrees. Some victims confirmed the situation led them to move house or even city, and others stated the situation was not very important and that they suffered only a few months after the occurrence.

Moreover, we found that the degree of impact is related to the duration and frequency of the SV, as well as the behaviors used by the perpetrator. Therefore, several studies suggest the need to educate students and (re)educate professionals, in order to make them aware of this problem, aiming toward greater prevention and, consequently, better intervention. In addition, some victims provided recommendations to help end or, at least, minimize the problem, among which are the need for more information on medical procedures, as well as investment in research within hospitals, in order to avoid strategies such as deception by the perpetrator and the placement of cameras and/or recorders in offices.

Furthermore, informing the population about patients’ rights and ethical guidelines is just as important as (re)educating health professionals and students in these areas. This way it will be easier for victims to recognize a situation of SV and act on it.

## Conclusion

In conclusion, for future research, we suggest the exploration of more specific characteristics about the victims in the national context, as well as the characteristics of the perpetrators, namely how they perceive the problem of SV by health professionals.

## Data availability statement

The raw data supporting the conclusions of this article will be made available by the authors, without undue reservation.

## Ethics statement

The studies involving human participants were reviewed and approved by the University of Maia Ethics and Deontology Council. The patients/participants provided their written informed consent to participate in this study.

## Author contributions

MF, VS-G, AC, and DM contributed to the conception, design of the study, analysis, and interpretation of results. VG contributed to the data collection and drafted the manuscript preparation. All authors reviewed the results and approved the final version of the manuscript. All authors made substantial contributions to the conception and design of the work, to the acquisition, analysis, and interpretation of data; drafted the work and revised it critically; approved the version to be published; and agree to be accountable for all aspects of the work in ensuring that questions related to the accuracy or integrity of any part of the work are appropriately investigated and resolved.

## References

[B1] Abrahams N. Devries K. Watts C. Pallitto C. (2014). Worldwide prevalence of non-partner sexual violence: A systematic review. *Lancet* 383 1648–1654. 10.1016/S0140-6736(13)62243-6 24529867

[B2] Alpert J. L. Steinberg A. L. (2017). Sexual boundary violations: A century of violations and a time to analyze. *Psychoanal. Psychol.* 34 144–150.

[B3] APA (2018). *Ethical principles of psychologists and code of conduct.* Washington, DC: American Psychological Association.

[B4] Bachmann K. M. Bossi J. Moggi F. Stirnemann-Lewis F. Sommer R. Brenner H. D. (2000). Nurse–patient sexual contact in psychiatric hospitals. *Arch. Sex. Behav.* 29 335–347. 10.1023/A:1001914303435 10948723

[B5] Barth J. Bermetz L. Heim E. Trelle S. Tonia T. (2013). The current prevalence of child sexual abuse worldwide: A systematic review and meta-analysis. *Int. J. Public Health* 58 469–483. 10.1007/s00038-012-0426-1 23178922

[B6] Blechner M. J. (2014). Dissociation among psychoanalysts about sexual boundary violations. *Psychoanalysis* 50 23–33.

[B7] Borys D. Pope K. (1989). Dual relationships between therapist and patient: A national study of psychologists, psychiatrists and social workers. *Prof. Psychol. Res. Pract.* 20, 282–293. 10.1037/0735-7028.20.5.283

[B8] Costa de Souza F. B. Drezett F. Meirelles A. C. Ramos D. G. (2013). Aspetos psicologógicos de mulheres que sofrem violência sexual [Psychological aspects of women who suffer sexual violence]. *Reprod. Clim.* 27 98–103.

[B9] Dahlberg C. C. (2014). Sexual contact between patient and therapist. *Contemp. Psychoanal.* 50 5–22. 10.1080/00107530.2014.868175

[B10] De Freitas M. L. Farinelli C. A. (2016). As consequências psicossociais da violência sexual [The psychosocial consequences of sexual violence]. *Rev. Em Pauta* 14 270–295.

[B11] Drezett J. (2014). Violência sexual contra a mulher e impacto sobre a saúde sexual e reprodutiva [Sexual violence against women and impact on sexual and reproductive health]. *Rev. De Psicol. Da UNESP* 2 36–50.

[B12] DuBois J. M. Walsh H. A. Chibnall J. T. Anderson E. E. Eggers M. R. Fowose M. (2019). Sexual violation of patients by physicians: A mixed-methods, exploratory analysis of 101 cases. *Sex. Abuse* 31 503–523. 10.1177/1079063217712217 28627296 PMC6031470

[B13] Eichenberg C. Becker-Fischer M. Fischer G. (2010). Sexual assaults in therapeutic relationships: Prevalence, risk factors and consequences. *Health* 2 1018–1026. 10.4236/health.2010.29150

[B14] Faulkner C. Regehr C. (2011). Sexual boundary violations committed by female forensic workers. *J. Am. Acad. Psychiatry Law* 39 154–163. 21653256

[B15] Fávero M. Carvalho J. Ferreira F. (2010). “Sexual aggression by professionals and boundary violations,” in *Paper presented at the oral presentation at the 10th congress of the european federation of sexology*, Porto.

[B16] Fávero M. Del Campo A. Faria S. Moreira D. Ribeiro F. Sousa-Gomes V. (2022). Rape myth acceptance of police officers in Portugal. *J. Interpers. Viol.* 37 659–680. 10.1177/0886260520916282 32306843

[B17] Fávero M. Moreira D. Abreu B. Del Campo A. Moreira D. S. Sousa-Gomes V. (2021). Psychological intervention with adult victims of sexual abuse: A comprehensive review. *Clin. Psychol. Psychother*. 29 62–80. 10.1002/cpp.2598 33844370

[B18] Fronek P. Kendall M. Ungerer G. Malt J. Eugarde E. Geraghty T. (2009). Towards healthy professional-client relationships: The value of an interprofessional training course. *J. Interprof. Care* 23 19–29. 10.1080/13561820802491006 19142780

[B19] Galletly C. A. (2004). Crossing professional boundaries in medicine: The slippery slope to patient sexual exploitation. *Med. J. Aust.* 181 380–383. 15462658 10.5694/j.1326-5377.2004.tb06334.x

[B20] Garrett T. (2002). “Inappropriate therapist-patient relationships,” in *Inappropriate relationships*, eds Goodwin R. Cramer D. (Mahwah, NJ: Erlbaum), 147–170.

[B21] Gartrell N. K. Milliken N. Goodson W. H. Thiemann S. Lo B. (1992). Physician-patient sexual contact prevalence and problems. *West. J. Med.* 157 139–143. 1441462 PMC1011231

[B22] Gewirtz-Meydan A. Finkelhor D. (2019). Sexual abuse and assault in a large national sample of children and adolescents. *Child Maltreat.* 25 203–214. 10.1177/1077559519873975 31526040

[B23] Gonçalves D. M. Stein A. T. Kapczinski F. (2008). Avaliação de desempenho do Self-Reporting Questionnaire como instrumento de rastreamento psiquiátrico: Um estudo comparativo com o Structured Clinical Interview for DSM-IV-TR. [Performance evaluation of the Self-Reporting Questionnaire as a psychiatric screening tool: A comparative study with the Structured Clinical Interview for DSM-IV-TR]. *Cad. Saúde Pública* 24 380–390. 10.1590/S0102-311X2008000200017 18278285

[B24] Gutheil T. G. Gabbard G. O. (1993). The concept of boundaries in clinical pratice: Theoretical and risk-magagement dimensions. *Am. J. Psychiatry* 150 188–196. 10.1176/ajp.150.2.188 8422069

[B25] Hook J. Devereux D. (2018). Boundary violations in therapy: The patient’s experience of harm. *Bjpsych. Adv.* 24 1–8. 10.1192/bja.2018.26

[B26] Hudgins K. (2022). Transformación del Triángulo del Trauma – TSM [Transformation of the Trauma Triangle]. *La Hoja De Psicodrama* 74 6–19.

[B27] Krahé B. (2016). “Societal responses to sexual violence against women: Rape myths and the “real rape” stereotype,” in *Women and children as victims and offenders: Background, prevention, reintegration*, eds Kury H. Redo S. Shea E. (Berlin: Springer), 10.1007/978-3-319-08398-8_24

[B28] Lazarus A. A. (1994). How certain boundaries and ethics diminish therapeutic effectiveness. *Ethics Behav.* 4 255–261. 10.1207/s15327019eb0403_10 11652796

[B29] Lovibond P. F. Lovibond S. H. (1995). The structure of negative emotional states: Comparison of the depression anxiety stress scales (DASS) with the beck depression and anxiety inventories. *Behav. Res. Therapy* 33, 335–343. 10.1016/j.rbp.2012.05.003 7726811

[B30] Melville-Wiseman J. (2012). Professional sexual abuse in mental health services: Capturing practitioner views of a contemporary corruption of care. *Soc. Work Soc. Sci. Rev.* 15 26–43. 10.1921/095352212X655320 36012862

[B31] Moher D. Liberati A. Tetzlaff J. Altman D. Group P. (2010). Preferred reporting items for systematic reviews and meta-analyses: The PRISMA statement. *Int. J. Surg.* 8 336–341. 10.1016/j.ijsu.2010.02.007 20171303

[B32] Moreira D. Moreira D. S. Barbosa F. Sousa-Gomes V. Fávero M. (2021). Childhood traumatic experiences and psychopathy: A comprehensive review. *Psychol. Trauma* [Epub ahead of print]. 10.1037/tra0001191 34843347

[B33] Pais-Ribeiro J. L. Honrado A. Leal I. (2004). Contribuição para o estudo da adaptação portuguesa das escalas de ansiedade, depressão e stress (EADS) de 21 itens de Lovibond e Lovibond [Contribution to the study of the Portuguese adaptation of the 21-item scales for anxiety, depression and stress of Lovibond and Lovibond (EADS)]. *Psicol. Saúde Doenças* 5 229–239.

[B34] Pope K. S. Levenson H. Schover L. (1979). Sexual intimacy in psychology training: Results and implications of a national survey. *Am. Psychol.* 34, 682–689. 10.1037//0003-066X.34.8.682 496089

[B35] Plaut S. M. (1997). Boundary violations in professional-client relationships: Overview and guidelines for prevention. *Sex. Marital Ther.* 12 77–94. 10.1080/02674659708408203

[B36] Plaut S. M. (2008). Sexual and nonsexual boundaries in professional relationships: Principles and teaching guidelines. *Sex. Relationsh. Ther.* 23 85–94. 10.1080/14681990701616624

[B37] Pope K. (1994). *Sexual involvement with therapists.* Washington, DC: American Psychological Association.

[B38] Reamer F. G. (2003). Boundary issues in social work: Managing dual relationships. *Natl. Assoc. Soc. Work.* 48 121–133. 10.1093/sw/48.1.121 12564713

[B39] Reling T. Becker S. Drakeford L. Valasik M. (2018). Exploring the influence of hookup culture on female and male rape myths. *J. Interpers. Viol.* 36 1–25. 10.1177/0886260518801021 30246612

[B40] Sansone R. A. Sansone L. A. (2009). Crossing the line: Sexual boundary violations by physicians. *Psychiatry* 6 45–48. 19724761 PMC2720840

[B41] Schoener G. R. Milgrom J. H. Gonsiorek J. C. Luepker E. T. Conroe R. M. (1989). *Psychotherpists’ sexual involvement with clients: Intervention and prevention.* Minneapolis, MN: Walk-In Counseling Center.

[B42] Smith D. Fitzpatrick M. (1995). Patient-therapist boundary issues: An integrative review oh theory and research. *Prof. Psychol.* 26 499–506. 14621713 10.1037/0735-7028.26.5.499

[B43] Sousa-Gomes V. Abreu B. Moreira D. Del Campo A. Moreira D. S. Fávero M. (2022). Psychological intervention and treatment programs for adult victims of child sexual abuse: A systematic review. *Psychol. Trauma.* 10.1037/tra000138 36301296

[B44] Ventura I. (2018). *Medusa no Palácio da Justiça ou uma história da violação sexual [Medusa in the Palace of Justice or a story of rape]. Tinta da China.* Ph.D thesis. Braga: Universidade do Minho.

[B45] Wies J. Coy K. (2013). Measuring violence: Vicarious trauma among sexual assault nurse examiners. *Hum. Organ.* 72 23–30. 10.17730/humo.72.1.x5658p957k5g7722

[B46] World Health Organization [WHO] (2002). *World Report on Violence and Health*. Geneva: World Health Organization.

